# Transient Sensorineural Hearing Loss and Rhabdomyolysis, Associated With Chronic Kratom Use

**DOI:** 10.1155/crot/9699246

**Published:** 2026-08-26

**Authors:** Hiroto Watanabe, Kathy Zhang, Yekaterina Shapiro

**Affiliations:** ^1^ Cooper Medical School of Rowan University, Camden, New Jersey, USA, rowan.edu; ^2^ Division of Otolaryngology—Head & Neck Surgery, Cooper University Health Care, Camden, New Jersey, USA

## Abstract

Kratom is a colloquial name for products derived from the mitragyna plant species originating in Southeast Asia and now widely available in the United States as a dietary supplement. Kratom is used primarily for self‐management of pain, anxiety, and opioid withdrawal due to its partial agonist activity on mu opioid receptors, as well as other neurotransmitter pathways. We present a case of a 32‐year‐old male who presented with septic shock requiring vasopressors and unilateral acute hearing loss. He was found to have acute kidney injury secondary to rhabdomyolysis and auriculitis. He required emergent hemodialysis for severe electrolyte abnormalities and treatment with antibiotics and steroids for auriculitis. He spontaneously recovered hearing during his hospital admission, and his kidney function improved. This case underscores the potential complications associated with kratom use and its sedating effects and the need for further characterization of kratom toxicity to distinguish it from other toxidromes.

## 1. Introduction

**FIGURE 1:**
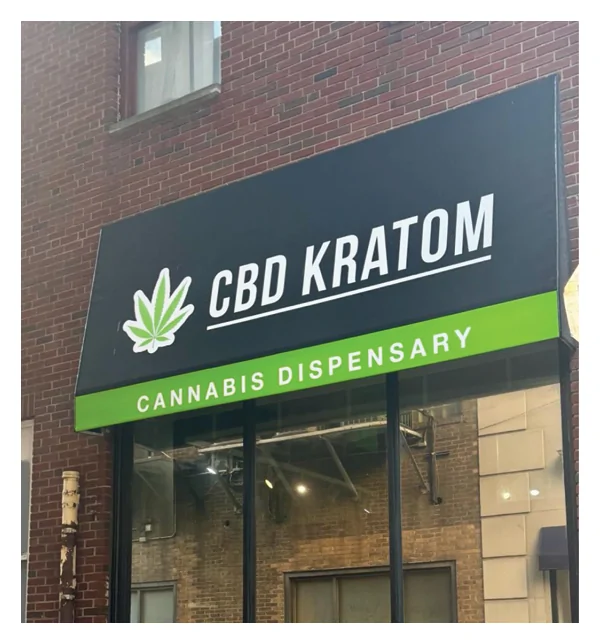
Photo of a cannabis shop in Philadelphia, PA, advertising kratom availability.

**FIGURE 2:**
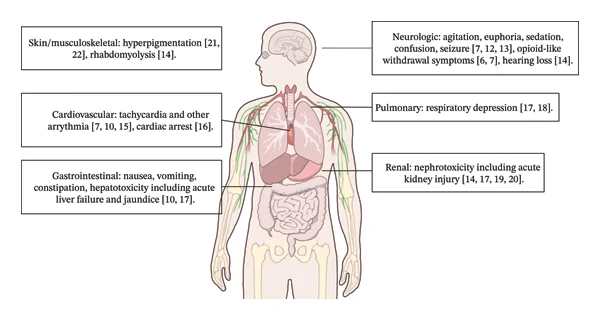
Summary of documented effects and associations of kratom on various organ systems.

Kratom, or mitragyna speciosa, is a tropical plant indigenous to Southeast Asia with opioid and stimulant‐like effects and was estimated to be used recreationally by up to 9.1% of the US population in 2024, and the number of kratom‐related reports to the National Poison Center has increased from 2015 to 2025 [1, 2]. Multiple alkaloid compounds can be isolated from kratom, including mitragynine and 7‐hydroxymitragynine, which have affinity for the mu opioid receptor [3]. The Food and Drug Administration (FDA) classifies kratom as an unapproved, high‐risk botanical substance with no legal use as a dietary supplement. Given its status as a botanical substance, it is not subject to the same level of regulatory scrutiny as medications or scheduled controlled substances and, therefore, the precise contents of legally purchased kratom have more potential to vary or to be adulterated to increase addictive potency [4]. However, kratom is not legal in all states, and there has been an increasing state‐level regulation [5]. The majority of individuals who use kratom are between the ages of 31 and 50 [6]. It can be purchased online, at smoke shops (Figure 1), or in gas stations in various forms such as teas, powders, capsules, and extracts [7]. Commonly used for its euphoric effects, some individuals also use kratom for anxiety, pain management, and to alleviate opioid and other illicit drug withdrawal [6, 8]. Typical doses of kratom are in the range of 2–6 g. Doses below 5 g are reported to have more stimulating effects, doses between 5 and 15 g to have opioid‐like effects, and doses greater than 15 g to have sedating effects [9]. A wide range of adverse effects reported from kratom use includes hypertension, tachycardia, hepatotoxicity [10], rhabdomyolysis, renal failure, and death [9, 11] (Figure 2 and Table 1). One reported overdose requiring patient intubation recorded a urine 7‐hydroxymitragynine level of > 500 ng/mL, and a fatal kratom accidental overdose has been recorded at a serum mitragynine level of 1.9 mg/L [11]. Currently, the exact mechanism of kratom toxicity is unknown, but a study in rats hypothesized that kratom toxicity causes injury to the kidney, liver, and lung [17].

**TABLE 1:** Summary of documented effects and associations of kratom on various organ systems.

Neurologic	Agitation, euphoria, sedation, confusion, seizure [7, 12, 13], opioid‐like withdrawal symptoms [6, 7], hearing loss [14]
Cardiovascular	Tachycardia and other arrythmia [7, 10, 15], cardiac arrest [16]
Pulmonary	Respiratory depression [17, 18]
Gastrointestinal	Nausea, vomiting, constipation, hepatotoxicity including acute liver failure and jaundice [10, 17]
Renal	Nephrotoxicity including acute kidney injury [14, 17, 19, 20].
Skin/musculoskeletal	Hyperpigmentation [21, 22], rhabdomyolysis [14]

The literature on the known otologic and audiologic consequences of opioids is currently growing and primarily focuses on case reports [23, 24]. Opioids, which share a receptor with kratom metabolites, have been associated with peripheral vestibular dysfunction, hearing loss of variable severity and laterality, and tinnitus in multiple different opioids, which may be further worsened when combined with stimulants [23–25]. One specific example includes a case series of hydrocodone/acetaminophen‐associated sensorineural hearing loss [26]. Cases of kratom‐associated kidney injury, including elevated with creatinine kinase [14, 19, 20], have previously been reported, with one case associated with concurrent hearing loss [14]. In that case, the patient had kratom toxicity with rhabdomyolysis secondary to compartment syndrome requiring a fasciotomy, complicated by heart failure with reduced ejection fraction and transient hearing loss which resolved with hemodialysis [14].

Given the dearth of other cases in the literature reporting on kratom ingestion and associated hearing loss, we present a case of sudden sensorineural hearing loss (SSNHL) in a 32‐year‐old male who presented with acute kidney injury (AKI) secondary to rhabdomyolysis following kratom use. As we were unable to contact the patient for consent despite multiple attempts, this case report has been maximally anonymized. Our institution does not require IRB consent for case reports.

## 2. Case Presentation

A 32‐year‐old with a past medical history of anxiety presented to an outside hospital emergency department with primary concern for right ear pain associated with right‐sided hearing loss. The patient was noted to have slurred speech and was initially unable to recall the events leading to presentation. He later noted he initially felt unsteady and woke up with a headache, nausea, right ear pain, and right‐sided hearing loss after being down for at least 8 h. He reported slipping and falling in the shower and waking up on the ground. He underwent a computed tomography (CT) head without contrast, which revealed a left occipital and right parietal scalp hematoma and no evidence of skull fractures. An initial set of labs showed hyponatremia to 131 mmol/L, hypocalcemia to 6.8 mg/dL, hyperkalemia to 7.0 mmol/L, hyperphosphatemia to 5.4 mg/dL, elevated BUN to 51 mg/dL, elevated aspartate aminotransferase to 1004 U/L and alanine aminotransferase to 355 U/L, creatinine of 3.98 mg/dL, and elevated creatine kinase (CK) to 90,665 U/L, consistent with AKI and rhabdomyolysis.

He reported taking 6–8 capsules of kratom (of unknown dosage) per day to self‐treat his anxiety for the past 2 years. He reported purchasing kratom from his gas station and disclosed that he had recently increased his kratom dosing but did not know the exact increase. He denied use of any other medications or substances including over the counter, herbal supplements, or illicit drugs.

On presentation to the outside hospital, he was reported to be in septic shock, with a lactate of 3.4 mmol/L and started on 6 mcg of norepinephrine as well as cefepime, vancomycin, and metronidazole. On arrival at our facility, blood pressure was 112/71, heart rate of 77, oral temperature of 97.5°F, and lactate of 1.2 mmol/L. Antibiotics were narrowed to cefazolin due to concern for auriculitis, and norepinephrine was stopped. Blood cultures were found to be without growth for 5 days. Urine drug screen (UDS) was negative for phencyclidine, barbiturates, benzodiazepines, cocaine, methadone, opiates, tetrahydrocannabinol, and amphetamines and positive for fentanyl. Additionally of note, fentanyl was not a documented medication given at either our hospital or the transferring hospital. Physical exam revealed right postauricular swelling and tenderness, right tragal tenderness, and diffuse right auricular erythema and tenderness including the ear lobe, consistent with auriculitis. However, a CT temporal bones was not performed during the admission due to review of a CT head on admission. On otoscopy, bilateral tympanic membranes were intact and there was no evidence of fluid in the middle ear spaces. A 512‐Hz tuning fork‐Weber test was inconclusive while the Rinne test was normal, showing air conduction greater than bone conduction.

For the first 2  days of his hospitalization in the neurological intensive care unit (ICU), his primary treatments consisted of cefazolin for auriculitis, aggressive fluid resuscitation for rhabdomyolysis, as well as calcium gluconate, insulin with dextrose, patiromer, and furosemide for hyperkalemia. Given his report of sudden onset hearing loss upon waking and medical instability precluding the ability to obtain a baseline audiogram, he was treated with empiric high dose steroids (prednisone 40 mg) twice daily beginning on Day 3 of admission in accordance with guidelines for treatment of SSNHL [27]. Magnetic resonance imaging (MRI) of the internal auditory canal (IAC) was ordered but initially delayed due to his medical instability.

He was downgraded from the ICU to general medicine floors on Day 3 and started his first of three intermittent hemodialysis sessions for AKI secondary to rhabdomyolysis. Electrolytes, creatinine, and creatinine kinase all improved with hemodialysis. On Day 5 of admission, he reported improvement in both his hearing and tenderness of the mastoid and tragus. On Day 6, he was medically stable enough to obtain an MRI to evaluate the etiology of his hearing loss. MRI IAC revealed soft tissue swelling and a rim‐enhancing fluid collection measuring approximately 2 cm, concerning for abscess within the soft tissues at the base of the right ear and nonspecific left posterior temporal occipital soft tissue swelling. He continued the antibiotic regimen of intravenous, cefazolin, 1 g, q12 h. On Day 10, an audiogram was obtained, which showed normal hearing bilaterally at 5–10 dB at all frequencies and Type A tympanograms bilaterally. On Day 10 of hospitalization, he was discharged home with a prednisone taper and transitioned to oral cephalexin. His creatinine did not return to baseline at discharge, 2.66 mg/dL, but was greatly improved from its maximum value of 6.1 mg/dL (Day 3 of hospitalization). Repeat outpatient labs 1 week after discharge revealed that creatinine levels had returned to baseline at 1.12 mg/dL.

## 3. Discussion

SSNHL is characterized by progression of hearing impairment of 30 dB or more with onset occurring within 72 h, often occurring unilaterally [28]. Although the causes of SSNHL can be multifactorial, many cases are idiopathic without an identifiable cause; infection, vascular impairment, autoimmune disorders, trauma, ototoxic substances, and central nervous system disease have been implicated in some cases [28]. Only one other case report of kratom use associated with hearing loss was found in the literature [14], while another case reported improvement of hearing loss secondary to Meniere disease with the use of kratom [29]. The effect of kratom on hearing is not well studied, including potential mechanisms and doses at which these effects may occur. The effects of hearing loss may be secondary to other physiologic pathway disruptions, other organ toxicity, or potentially the contamination of kratom products with ototoxic substances.

Rhabdomyolysis is a life‐threatening condition characterized by dissolution of skeletal muscles and leakage of muscle cell contents into circulation. Rhabdomyolysis can be caused by muscle injury in the context of trauma or exercise or lack of adenosine triphosphate such as in metabolic disorders and ischemia. Pressure‐induced muscle injury, such as when a person is immobilized on a hard surface and does not intermittently shift their body weight [30, 31], is one scenario that may cause rhabdomyolysis. We suspect this occurred in our patient due to sedating effects of either kratom or fentanyl from his initial UDS. These sedating effects were also likely a factor leading up to the patient’s fall and scalp hematomas. AKI is one sequela of rhabdomyolysis that may require hemodialysis when aggressive fluid resuscitation alone is unable to improve the AKI [32]. The mechanism of the few reported cases of rhabdomyolysis associated with kratom [14, 20, 33, 34] is suggested to be secondary to pressure‐induced muscle injury.

Electrolyte changes, which our patient suffered from as a result of the AKI, may have played a role in his hearing loss. Systemic hyperkalemia is hypothesized to disrupt the unique electrochemical gradient in the cochlea critical for normal hair cell depolarization [35] although one recent study did not find a significant association between potassium levels and sensorineural hearing loss (SNHL) [36]. Additionally, calcium is a key electrolyte for maintaining cochlear homeostasis [37] and one study found that hypocalcemia was an independent risk factor for development of SNHL in children [38]. On literature review, there is no specific serum calcium threshold established at which SNHL would be expected to occur.

While hypothesizing the role of kratom in the patient’s presentation, we must also consider how fentanyl may have affected the patient given the patient’s positive UDS. It is possible that fentanyl caused sedation, which resulted in his fall and the prolonged time on the bathroom floor, although the patient denied use of substances except for kratom. It is also possible that the screening UDS was a false positive [39]. Confirmatory testing for the positive fentanyl UDS was not performed as it was deemed that it would not change patient care. Use of opioids with kratom is of high concern given its noted potential for worse consequences than opioids alone, including higher incidence of death [2]. Kratom alkaloid metabolites are known inhibitors of the enzymes CYP2D6 and CYP3A, which may result in slower metabolization of some drugs, including fentanyl [40]. Opioids have also been associated with SSNHL in literature reviews and case reports although the exact mechanisms are not well understood. This includes oral hydrocodone/acetaminophen, morphine, illicit opioids such as heroin and the concomitant use of cocaine, alcohol, and benzodiazepines [23–26, 41–43]. Given kratom’s ability to slow opioid metabolism, it may be beneficial for clinicians to regularly ask about kratom use when obtaining a detailed history given the increase in kratom‐related calls to the poison control centers [2], especially when opioids are prescribed as treatment.

At this time, we are unable to decisively identify the cause of the patient’s transient hearing loss associated with AKI, rhabdomyolysis, postauricular abscess, hyperkalemia, hypocalcemia, and elevated transaminases, all of which improved with emergent hemodialysis, oral corticosteroids, and antibiotics. Given that our patient’s hearing improved to normal thresholds on audiometric testing after treatment, the nature of his hearing loss could have been idiopathic SSNHL with improvement on high‐dose steroids or might point toward the likely association between his AKI, electrolyte derangements, and rhabdomyolysis as potential contributors. Alternative explanations for this patient’s SSNHL and rhabdomyolysis could be toxicity from high‐dose kratom and its metabolites or contaminants. As he tested positive for fentanyl on UDS, it may have also been a driver for his presentation, either on its own or its toxicity amplified by the inhibition of CYP2D6 and CYP3A by kratom metabolites. While testing for kratom metabolites is currently not common, it may be helpful to increase access to testing of urine 7‐hydroxymitragynine level or serum mitragynine to more thoroughly investigate similar cases in the future. This case explores some of the potential complications of kratom toxicity, especially sedation, and further highlights the concern for the FDA to classify kratom as a scheduled substance.

## Funding

The authors received no funding for this work.

## Conflicts of Interest

The authors declare no conflicts of interest.

## Data Availability

The data that support the findings of this study are available on request from the corresponding author. The data are not publicly available due to privacy or ethical restrictions.
